# Treatment with etanercept and low monocyte concentration contribute to the risk of invasive aspergillosis in patients post allogeneic stem cell transplantation

**DOI:** 10.1038/s41598-019-53504-8

**Published:** 2019-11-21

**Authors:** Tamara Zoran, Michael Weber, Jan Springer, P. Lewis White, Joachim Bauer, Annika Schober, Claudia Löffler, Bastian Seelbinder, Kerstin Hünniger, Oliver Kurzai, André Scherag, Sascha Schäuble, C. Oliver Morton, Hermann Einsele, Jörg Linde, Jürgen Löffler

**Affiliations:** 1 University Hospital Würzburg, Medical Hospital II, WÜ4i Würzburg, Germany; 2 Friedrich Löffler Institute, Institute of Molecular Pathogenesis, Jena, Germany; 3 Leibniz Institute for Natural Product Research and Infection Biology–Hans Knöll Institute, Jena, Germany; 4 Public Health Wales, Microbiology, Cardiff, UK; 5 Septomics Research Centre, Friedrich Schiller University and Leibniz Institute for Natural Product Research and Infection Biology–Hans Knöll Institute, Jena, Germany; 6 Institute for Hygiene and Microbiology, University of Würzburg, Würzburg, Germany; 7 Institute of Medical Statistics, Computer and Data Sciences, University Hospital, Jena, Germany; 8 Friedrich Löffler Institute, Institute of Bacterial Infections and Zoonoses, Jena, Germany; 9 Western Sydney University, School of Science and Health, Campbelltown, NSW 2560 Australia

**Keywords:** Machine learning, Infectious-disease diagnostics, Diagnostic markers, Risk factors

## Abstract

Invasive aspergillosis (IA) is a life-threatening complication among allogeneic hematopoietic stem cell transplant (alloSCT) recipients. Despite well known risk factors and different available assays, diagnosis of invasive aspergillosis remains challenging. 103 clinical variables from patients with hematological malignancies and subsequent alloSCT were collected. Associations between collected variables and patients with (n = 36) and without IA (n = 36) were investigated by applying univariate and multivariable logistic regression. The predictive power of the final model was tested in an independent patient cohort (23 IA cases and 25 control patients). Findings were investigated further by *in vitro* studies, which analysed the effect of etanercept on *A. fumigatus*-stimulated macrophages at the gene expression and cytokine secretion. Additionally, the release of C-X-C motif chemokine ligand 10 (CXCL10) in patient sera was studied. Low monocyte concentration (p = 4.8 × 10^−06^), severe GvHD of the gut (grade 2–4) (p = 1.08 × 10^−02^) and etanercept treatment of GvHD (p = 3.5 × 10^−03^) were significantly associated with IA. Our studies showed that etanercept lowers CXCL10 concentrations *in vitro* and *ex vivo* and down-regulates genes involved in immune responses and TNF-alpha signaling. Our study offers clinicians new information regarding risk factors for IA including low monocyte counts and administration of etanercept. After necessary validation, such information may be used for decision making regarding antifungal prophylaxis or closely monitoring patients at risk.

## Introduction

Invasive aspergillosis (IA) is the most relevant mould infection in immunocompromised patients. It is associated with significant mortality in patients with haematological malignancies and those receiving allogeneic stem cell (alloSCT) or solid organ transplants^1–3^. Diagnosis of IA is challenging as clinical symptoms are often non-specific and conventional diagnosis is poor^4,5^. Methods such as high resolution computed tomography scans^6^ only show typical signs once infection is established, and even then, specific signs can be transient and could be associated with manifestations other than IA^4,7^. Serological tests detecting galactomannan (GM) and β-D-glucan have low positive predictive values and are better used for exclusion rather than for diagnosis of IA^4,5,7^. PCR-based assays to detect fungal DNA in patient specimens operate at the very limit of detection, due to the small amounts of fungal DNA recovered from blood samples and positivity occurs only transiently^8^. Although the testing of bronchoalveolar lavage fluid is likely associated with increased quantities of biomarker, sampling is often contra-indicated by the patient’s underlying clinical condition. As IA is a multi-factorial disease, it seems promising to combine different biomarkers, such as the use of GM and PCR assays to improve diagnosis^9–11^.

While there is limited evidence of current diagnostic assays being used to monitor the fungal load for prognostic purposes or during antifungal therapy, host biomarkers might better reflect the clinical course of IA. Furthermore, host biomarkers might allow individual pre-emptive risk stratification for IA. These markers include genetic (single nucleotide polymorphisms (SNPs)), clinical, environmental, and personal factors, which determine the individuals risk for IA and potentially the course and outcome of IA^12,13^. They include among others advanced age, the type of underlying disease, the dose and duration of corticosteroid therapy, viral infection (e.g. cytomegalovirus (CMV) or respiratory virus infection), lymphopenia, and neutropenia^13,14^. Although many of these factors have been individually associated with IA, defining patient-specific risk profiles for IA remains extremely challenging, as knowledge about the combination of risk factors with and without fungal biomarkers remains scarce.

The aim of this study was to identify distinct new risk factors, which in combination with known risk factors and fungal biomarkers might allow risk stratification and improve diagnosis of IA in patients that underwent alloSCT. Therefore, our approach is to identify significant associations between a large number of individual clinical variables and the occurrence of IA in parallel to identify new significant risk factors for the development of IA after alloSCT. Such risk factors might in future allow the definition of personalized stratification protocols for alloSCT patients according to their individual risk of developing IA.

**Figure 1:**
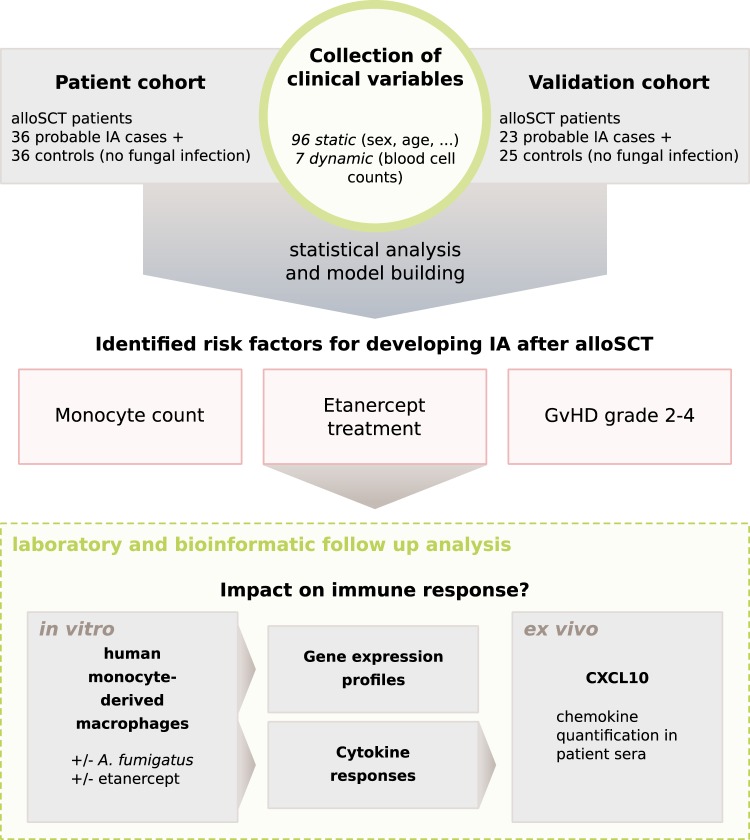
Overall study design and workflow. Clinical variables from allogeneic hematopoietic stem cell transplant (alloSCT) recipients were collected after stem cell transplantation twice weekly, whenever feasible. Monocyte count and treatment with etanercept were confirmed in validation cohort and therefore studied further.

We present a longitudinal case-control study, where post stem cell transplantation clinical variables were collected twice weekly, when feasible (Fig. 1). We compared 36 alloSCT patients with probable IA and 36 alloSCT controls with no evidence of fungal infection. The relevance of variables identified in this study as significantly associated with IA was validated in a confirmatory study of 23 cases of probable IA and 25 control alloSCT patients.

To support and interpret our findings, the function of the Tumor Necrosis Factor-alpha (TNF-alpha) blocker etanercept, which has been also used to treat chronic and acute graft-versus-host disease (GvHD), was studied on cytokine secretion and gene expression profiles were investigated *in vitro* in monocyte-derived macrophages stimulated with *Aspergillus fumigatu*s. Furthermore, CXCL10 release in serum samples from probable IA patients with or without etanercept treatment was investigated.

## Results

We performed a case-control study, consisting of 36 patients suffering from probable IA and 36 control patients without any signs of fungal infection after alloSCT. Detailed information regarding patient demographics is shown in Materials and Methods (paragraph “selection of patients”) and in Table 1. From these patients, we collected time-resolved data related to 96 binary clinical and seven continuous variables.

**Table 1:** Clinical details of the collected patient data including age, underlying disease, stem cell transplantation donor and type of antifungal treatment.

	Patients without IA	Patients with probable IA	All patients
**total number**	36	36	72
**age (median, range)**	54 (21–72)	56 (20–69)	55 (20–72)
**gender, male**	24	23	47
**gender, female**	12	13	25
**underlying disease**			
**acute myeloid leukemia**	7	13	20
**myelodysplastic syndrome**	4	4	8
**acute lymphoblastic leukemia**	2	5	7
**chronic lymphocytic leukemia**	2	3	5
**multiple myeloma**	11	4	15
**other diseases**	10	7	17
**donor source**			
**matched unrelated donor**	26	21	47
**matched related donor**	10	15	25
**mold-active therapy in detail**	17	30	47
voriconazole	10	17	27
posaconazole	8	13	21
caspofungin	6	7	13
anidulafungin	4	2	6
amphotericin B	3	8	11
**fluconazole**	34	29	63

Among them, seven binary variables were significantly associated with IA (p < 0.05, Table 2). However, positive Galactomannan (GM) ELISA and *Aspergillus* PCR test results in patients with probable IA do not represent independent variables as they are linked to the diagnosis of IA^6,15^. The use of nitroimidazole and dexamethasone and the presence of *Enterococcus faecalis* in the blood culture could not be confirmed in the validation cohort as an independent risk factor. Thus, we excluded these variables from the following studies and focused our further analyses and functional studies on the two remaining significant variables, more precisely the administration of etanercept and GvHD of the gut (grade ≥ 2). In the next step, we screened the specified continuous variables for association with IA.

**Table 2:** Clinically significant variables associated with diagnosis of invasive aspergillosis.

Variables	Nr. of cases (m:f)	Nr. of controls (m:f)	OR (95% CI)	p-value
galactomannan	28 (11:17)	3 (0:3)	38.5 (10.5 to 192.8)	3.10 × 10^−10^
*Enterococcus faecalis*	11 (5:6)	1 (0:1)	15.4 (2.7 to 290.6)	7.20 × 10^−04^
etanercept	13 (6:7)	3 (2:1)	6.2 (1.8 to 29.4)	3.50 × 10^−03^
dexamethasone	17 (8:9)	6 (3:3)	4.5 (1.6 to 14.3)	4.70 × 10^−03^
GvHD of the gut (grade ≥2)	13 (5:8)	4 (3:1)	4.5 (1.4 to 17.7)	1.08 × 10^−02^
nitroimidazole	22 (8:14)	12 (3:9)	3.1 (1.2 to 8.5)	1.70 × 10^−02^
*Aspergillus* PCR	15 (6:9)	8 (3:5)	4.0 (1.2 to 14)	2.00 × 10^−02^

For each clinical variables number of cases and controls (nr.), proportion of males and females (m:f), odds-ratio (OR) and likelihood ratio p-value are reported.

### Low monocyte concentration is a quantitative risk factor for IA after alloSCT

Temporally continuous variables, including the blood counts of various types of leukocytes were investigated over a period of 4 weeks prior to the diagnosis of IA. One week before diagnosis the concentration of total leukocytes, monocytes and granulocytes were all significantly associated with the diagnosis of IA (Table 3). The total leukocyte concentration was significantly reduced to 611 cells/µl in cases in contrast to 1101 cells/µl in controls (odds ratio (OR) = 1.14, 95% confidence interval (CI) = 1.1 to 1.3, p = 1.4 × 10^−3^), while granulocyte concentration reached levels of 354 cells/µl (cases) and 648 cells/µl (controls) (OR = 1.33, 95% CI = 1.1 to 1.7, p = 4.1 × 10^−03^) (Table 3).

**Table 3:** Blood cell concentration of monocytes, leukoytes and granulocytes were analysed by univariate general linear models for each week before diagnosis.

Cell type	Week before IA	Case	Control	OR (95% CI)	p-value
monocytes	1	0.44	1.62	13.3 (3.3 to 80.2)	4.8 × 10^−06^
	2	0.44	1.14	3.9 (1.4 to 18.9)	6.2 × 10^−03^
	3	0.32	0.88	11.9 (2 to 134)	2.7 × 10^−03^
	4	0.24	1.62	589.0 (31 to 52497)	1.1 × 10^−09^
leukocytes	1	6.11	11.01	1.1 (1.1 to 1.3)	1.4 × 10^−03^
	2	5.18	9.61	1.0 (1.02 to 1.2)	1.1 × 10^−02^
	3	4.53	9.03	1.1 (1 to 1.2)	8.7 × 10^−03^
	4	6.71	11.8	1.1 (1 to 1.15)	4.0 × 10^−02^
granulocytes	1	3.54	6.48	1.3 (1.1 to 1.7)	4.1 × 10^−03^
	2	3.11	5.77	1.2 (0.98 to 1.65)	0.1
	3	2.44	5.90	1.3 (0.99 to 2)	5.5 × 10^−02^
	4	1.09	26.78	6.4 (2.2 to 49)	2.2 × 10^−07^

For each model average concentration (x 10^2^ per micro-liter), odds-ratio (OR) and likelihood ratio p-value are reported.

**Figure 2:**
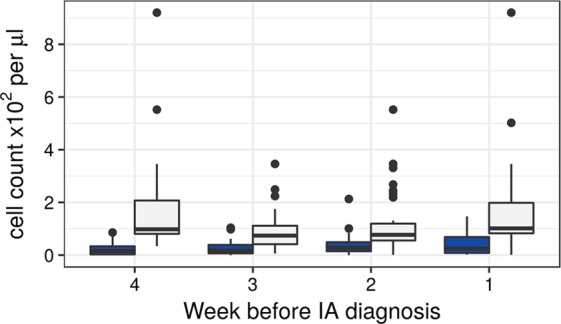
Boxplot showing concentrations of monocytes up to four weeks before IA diagnosis for cases. Control data (white boxplots) was matched to the time point of alloSCT of all cases (blue boxplots) per week. Differences were determined by the Likelihood-Ratio test. Nominally significant differences (i.e. p ≤ 0.05) were observed for all 4 weeks.

In particular, monocyte concentrations were relatively lowered in cases to 44 cells/µl compared to controls 162 cells/µl (OR = 13.26, 95% CI = 3.3 to 80.2, p = 4.8 × 10^−06^) (Fig. 2). The earlier weeks (up to four weeks prior to the diagnosis) exhibited a similar significant reduction of the monocyte concentration (Fig. 2). In validation data, we observe significantly lowered monocytes, but only starting at second week before diagnosis (OR = 4.97, 95% CI = 1.23 to 32.19, p = 0.018), whereas the first week before diagnosis shows no changes in monocyte concentration.

### Graft versus Host Disease of the gut (grade ≥ 2) is a risk factor for IA

Severe GvHD of the gut (grade 2–4) was significantly associated with IA (OR = 4.52, 95% CI = 1.4 to 17.71, p = 1.08 × 10^−02^). Case patients developed GvHD with a risk of 36% (13/36), while controls exhibited a lower risk of 11% (4/36). To verify this result, we also tested the significance of GvHD in our validation cohort result (OR = 6.92, 95% CI = 1.51 to 50.24, p = 1.1 × 10^−02^). In comparison, a general GvHD variable which also included less severe types of other organs (e.g. skin) was not significantly associated with IA.

### Etanercept is a binary risk factor for IA after alloSCT

The TNF-alpha blocker etanercept, which is frequently used for treatment of autoimmune inflammatory diseases, is also used to treat severe refractory GvHD.

From the group of drug-linked variables, etanercept was significantly associated with IA (OR = 6.22, 95% CI = 1.8 to 29.4, p = 3.5 × 10^−03^). In fact, the probability of getting IA after alloSCT under etanercept treatment among cases was 36% (13/36) in contrast to 8% (3/36) in controls. To validate etanercept as a risk factor for IA after alloSCT, we performed our statistical analyses in the validation cohort, which showed that 80% (8/10) of the patients who received etanercept treatment developed IA (OR = 6.13, 95% CI = 1.32 to 44.5, p < 0.019). Interestingly, both, severe GvHD of the gut (grade 2–4) and the use of etanercept were significantly associated with IA in univariate models. Therefore, we tested the effect of etanercept, when added as a new variable to the GvHD model. Notably, the predictive power of the model was significantly increased (p = 0.0152). In fact, patients with etanercept treatment of severe GvHD showed the highest risk to develop IA (10/10, 100%), compared to a lower risk (3/7, 43%) for patients developing GvHD of the gut (grade ≥ 2), which were not treated with etanercept (Table 2).

### Combination of risk factors

A multiple logistic regression model combined the different selected variables into a single model. This allowed the prediction of disease status based on knowledge about GvHD, etanercept treatment in combination with the appearance of low monocyte counts.

**Figure 3:**
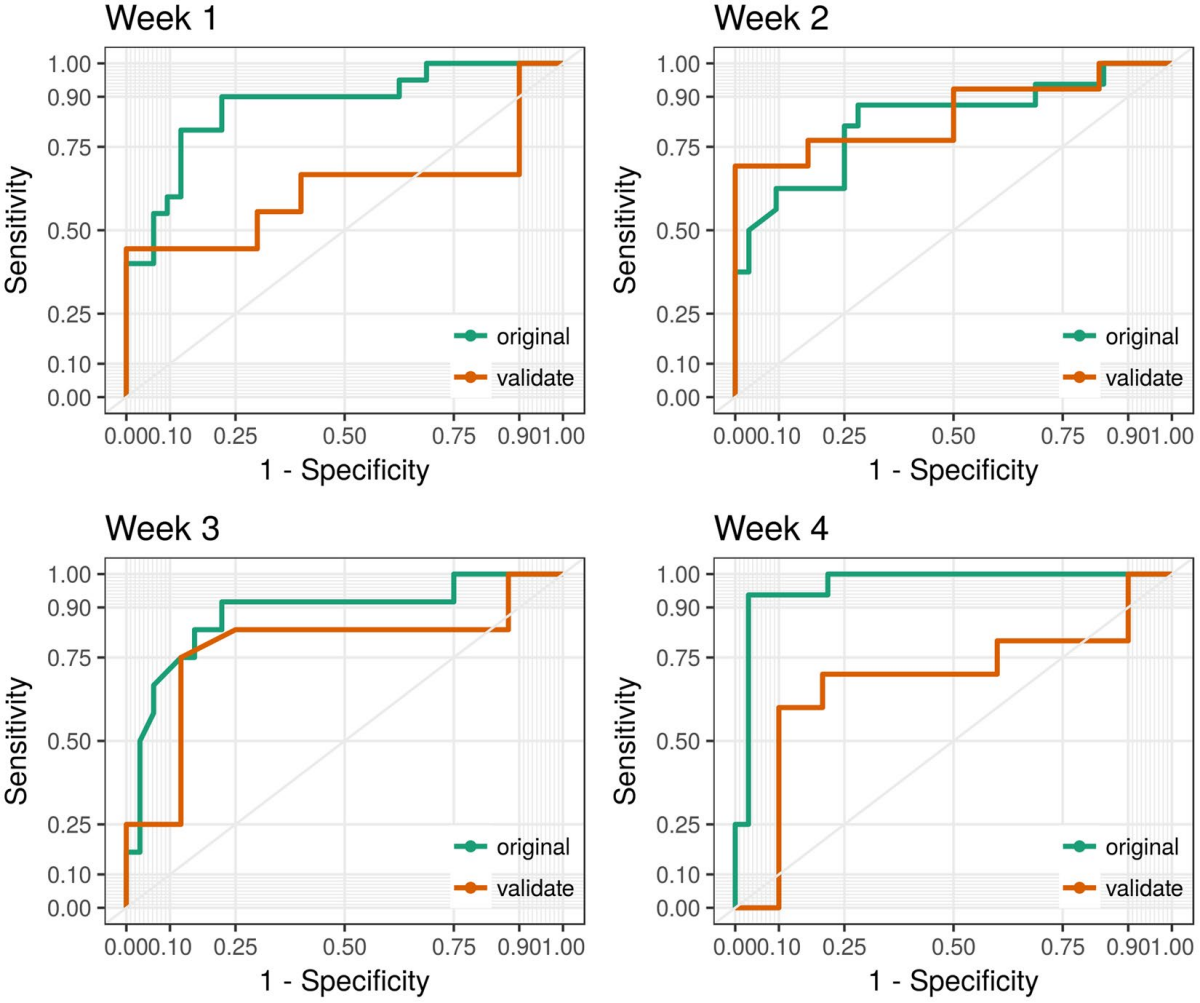
ROC curves show the application of the multivariable logistic regression models to each week separately for both original data and the validation data. Variables comprise etanercept and monocyte concentration.

As monocyte concentration significantly varied over time, we generated a model based on all data up to four weeks prior to the diagnosis of IA. For model evaluation, we applied the model subsequently on the validation data. Receiver operating characteristic (ROC) analysis was used to quantify the performance of the model. In Fig. 3, we show the resulting ROC curves for each week. The predicted diagnostic model performs exceptionally well on the validation data within week 2 (AUC = 0.85, 95% CI = 0.73 to 1) and 3 (AUC = 0.78, 95% CI = 0.54 to 1), while in week 1 (AUC = 0.62, 95% CI = 0.31 to 0.88) and week 4 (AUC = 0.68, 95% CI = 0.36 to 0.89) the model still provides a good classification. One explanation is that several cases appear to have elevated monocyte counts in week 1, which leads to lowered model sensitivity.

### The effect of etanercept on CXCL10 secretion

To identify and interpret any potential influence of etanercept on the immune response against *A. fumigatus*, we performed a variety of functional *in vitro* assays. Human primary MDM were selected, as they are among the most important cells involved in defense against IA. MDM were co-cultured with live *A. fumigatus* germ tubes in presence or absence of etanercept.

**Figure 4:**
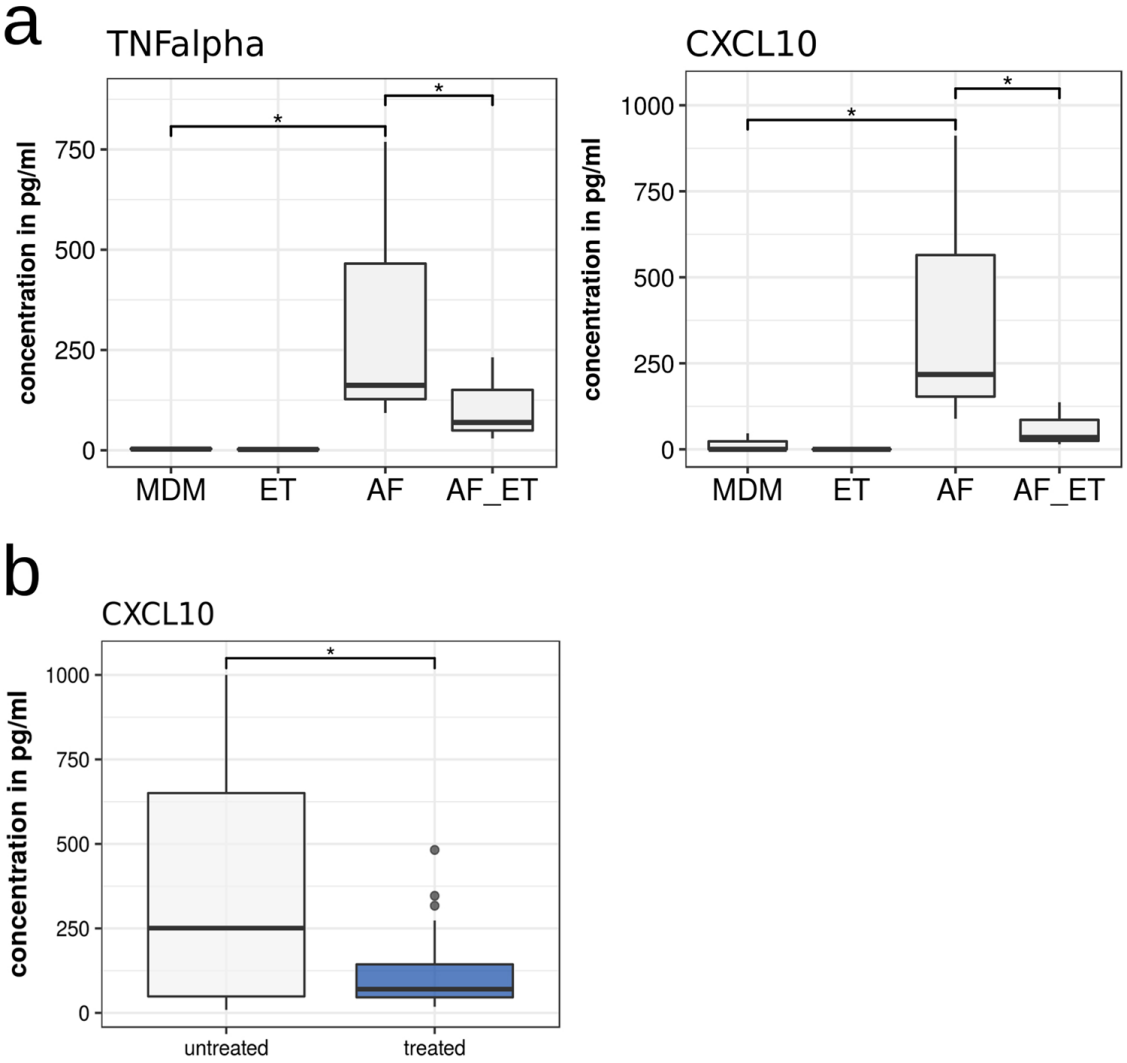
Comparison of cytokine release in monocyte-derived macrophages (MDM) stimulated with *A. fumigatus* in presence or absence of etanercept. Differences were determined by the Wilcoxon-Mann-Whitney test (p ≤ 0.05 are highlighted by *). (**a**) Secretion of cytokines TNF-alpha (p = 0.0104) and CXCL10 (p = 0.0004) is significantly downregulated in MDM stimulated with *A. fumigatus* under etanercept treatment (AF_ET) compared MDM stimulated with *A. fumigatus* only (AF). (**b**) CXCL10 release in serum samples from eight IA patients without etanercept treatment (untreated) and IA patients under etanercept treatment (treated).

As determined by multiplex ELISA analysis of the culture supernatants, confrontation of MDM with *A*. *fumigatus* induced a strong inflammatory response characterized by significant up-regulation of ten cytokines (Supplementary Table S2a). Treatment of unstimulated MDM with etanercept led to low secretion of IL-1RA and MCP-1 (Supplementary Table S2b). As expected, we detected lower TNF-alpha levels (p = 0.0104) after stimulation of MDM with *A. fumigatus* germ tubes in the presence of etanercept than compared to MDM which were infected with *A. fumigatus* germ tubes only (Fig. 4, Supplementary Table S2c). Interestingly, our data also showed a strong reduction of CXCL10 (IP-10) release (p = 0.0004) in etanercept-treated MDM stimulated with *A. fumigatu*s germ tubes (Fig. 4).

To validate this finding, CXCL10 levels in serum samples from 8 selected patients with probable IA were performed. Our data showed that patients with probable IA and etanercept treatment had significantly lower CXCL10 serum concentrations (p = 0.017) than patients without etanercept treatment (Fig. 4).

Furthermore, to achieve a more holistic view on the influence of etanercept on the macrophage – *A. fumigatus* interaction *in vitro*, we performed RNA-sequencing and compared gene expression of MDMs treated with etanercept and confronted with *A. fumigatus* germ tubes to gene expression of MDM confronted with *A. fumigatus* germ tubes only. Non-unstimulated MDM treated with etanercept served as a control. Confrontation of MDM with *A. fumigatus* germ tubes led to a significant induction of the expression of 382 genes while only 6 genes were repressed. *A. fumigatus* induces a fast and strong induction of genes involved in immune response and inflammation. Our data showed a more than 80-fold induction of the genes encoding for the chemokines CXCL2, CXCL3, CCL4 and CXCL10, respectively. Interestingly, etanercept treatment led to only minor transcriptional changes in unstimulated MDM. The drug did not significantly induce the expression of any gene while it repressed the expression of six genes encoding metallothioneins (*MT2A, MT1E, MT1X, MT1F, MT1H, MT1M*).

**Figure 5:**
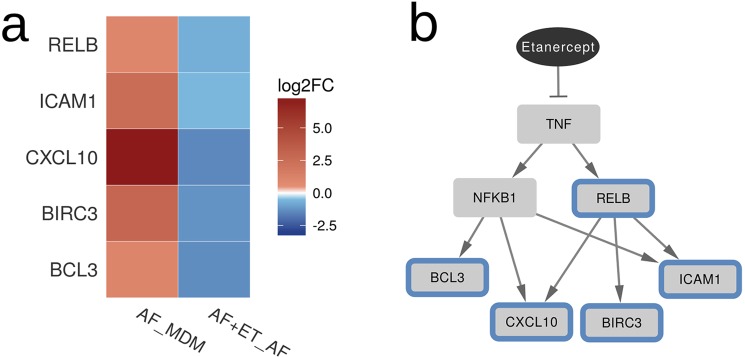
Regulation of NF-kappaB target genes which are differentially expressed in monocyte-derived macrophages treated with *A. fumigatus* and etanercept (AF + ET) compared to *A. fumigatus* (AF) only. Down-regulated genes are marked with blue color. (**a**) The heatmap displays downregulation of genes after addition of etanercept and the regulatory network (**b**) summarizes the effect of etanercept on the TNF-alpha signaling pathway.

Next, we compared gene expression profiles of MDM treated with etanercept and *A. fumigatus* germ tubes to gene expression profiles of MDM confronted with the fungus only. Among the 18 differentially expressed genes which were all down-regulated in MDM treated with etanercept and *A. fumigatus*, were genes encoding for metallothioneins (mentioned above) and genes encoding *CXCL10, DRAK1, ICAM1, BID, FUT4, RELB, RASGRP1, RNF144B, POU2F2, BIRC3, SLC2A6 and BCL3* (Fig. 5a, Supplementary Table S3). Although *TNF-alpha* expression was not significantly altered after etanercept treatment, genes located downstream of TNF-alpha including the transcription factor *RELB*, the *intercellular adhesion molecule 1* (*ICAM1*) and the apoptotic molecules *BCL3* and *BIRC3* were significantly downregulated in the presence of *A. fumigatus* and etanercept, compared to MDM stimulated with *A. fumigatus* only (Fig. 5).

## Discussion

Among opportunistic infections in immunocompromised patients, IA remains most challenging. The detection of fungal biomarkers in blood is imperfect due to variable sensitivity and specificity of the available diagnostic assays, especially in non-neutropenic patients or those receiving antifungal therapy or mold-active prophylaxis. Furthermore, positivity often occurs transiently as many diagnostic assays are run at the very limit of detection, due to the small amounts of fungal biomarkers. Consequently, and in contrast to many other infectious agents, following the fungal load for prognostic purposes or antifungal therapy monitoring is limited with the current diagnostic tools. Thus, the discovery and validation of new biomarkers are mandatory. This includes host biomarkers, which might better reflect the clinical course of IA and allow monitoring the response to antifungal therapy. Furthermore, host biomarkers might also allow risk stratification leading to an earlier start of antifungal treatment in afebrile patients thus resulting in more comprehensive pre-emptive treatment strategies.

Among the biomarkers described in this study, low monocyte counts up to four weeks prior to the diagnosis of probable IA was significantly associated with disease. Monocytes serve three main functions, including phagocytosis of microbes, the presentation of antigens to cells of the adaptive immune system, and the production of inflammatory cytokines and chemokines^16^. Moreover, monocytes also act as precursors for other inflammatory immune cells^17^. Therefore, reduction in monocyte levels might lead to a disrupted immune response. Our study revealed that monocytopenia occurred in patients with probable IA to a mean concentration of 44 cells/µl blood, compared to 162 cells/µl in control patients one week before diagnosis of IA (Fig. 2). These results imply that monocytopenia should be regarded as a risk factor for IA after alloSCT.

The high classification performance of the multivariable model on the validation data (validation cohort) underlines the predictive power of our variables (monocytes, GvHD and etanercept), while the temporal variation of the AUC performance remains a challenge.

Etanercept is a TNF-alpha blocker that antagonizes ligand binding and activation of tumor necrosis factor receptor 2 (TNFR2) and consequently inhibits downstream signaling including NF-κB activation. It can bind both, the soluble and the membrane form of TNF-alpha molecules removing them from the circulation^18^. TNF-alpha is a key cytokine involved in inflammatory and immune responses and has been shown to play a crucial role during fungal infections^19^. Thus, the use of drugs that block host immunity against microorganisms likely increases the risk of infection^19–22^. Since etanercept is most commonly used for the treatment of autoimmune diseases, many studies have focused on investigating mechanisms of action and side-effects of this drug among rheumatoid arthritis (RA) patients^22,23^. Etanercept has been already associated with an increased incidence of opportunistic infections in RA patients^19^, including bacterial and viral infections^20–22,24^.

Salvage therapy with high-dose corticosteroids and etanercept is used only in steroid-refractory GvHD with gastrointestinal involvement^25–27^. Until now, data on fungal infections associated with TNF-alpha blockade were limited to isolated cases or small number of patients. In a previous study from Tsiodras and colleagues^21^ it was shown that the most prevalent invasive fungal infections associated with TNF-alpha antagonist treatment were histoplasmosis (30%), candidiasis (23%) and aspergillosis (23%). This study focused on fungal infections associated with TNF-alpha blockade from 1966 to 2007 and revealed that among different patient groups receiving TNF-alpha blocker therapy, the GvHD group showed the worst clinical picture^21^. Although etanercept is available for the treatment of severe GvHD, little is known about its mechanism of action in association with *Aspergillus* spp. infections.

*In vitro* studies allow the reproduction of results observed *in vivo* by using highly controlled and standardized conditions. MDM represent a major TNF-alpha producing cell population of the innate immune system and play a major role in the immune defence against *A. fumigatus*. Confrontation of MDM with etanercept *in vitro* resulted in down-regulation of 6 genes that encode metallothioneins which are involved in the maintenance of zinc homeostasis^28^. By binding and releasing zinc, metallothioneins regulate zinc levels within the body, which influences the activation and binding of a variety of transcription factors. In addition, and as expected, stimulation of immune cells with *A. fumigatus* resulted in strong induction of genes involved in inflammatory responses^29,30^. Scicluna and colleagues^31^ reported that etanercept pre-treatment leads to a decreased transcriptional response to LPS. Furthermore, Tan and colleagues^23^ showed regulation of multiple inflammatory pathways in patients with psoriasis treated with etanercept. Data from our study showed the down-regulation of genes involved in immune responses and TNF-alpha signalling (Fig. 5). Significantly, down-regulated genes were involved in NFkB signalling, antimicrobial humoral response and apoptotic processes (*ICAM1, BCL3, BID, BIRC3, RELB, DRAK1* and *FUT4*) (Supplementary Table S3). For example, ICAM1 is an important adhesion molecule, expressed in immune cells^32^ and plays a major role in the immune response in association with T-cells^33^. BCL3 (B-cell lymphoma 3) is a regulator of the NFkB activity and essential for the proper development, survival and activity of adaptive immune cells^34^. *BIRC3* (Baculoviral IAP repeat-containing protein3) encodes cIAP2, which is an element of the TNFR2 receptor complex and therefore plays an important role in the TNF-alpha signalling pathway^35^. RELB (RELB Proto-Oncogene, NF-KB Subunit) is one of the members of the NFkB family and is activated by Dectin-1, which recognizes specific morphologies of *A. fumigatus*^36,37^.

Our *in vitro* studies showed that the presence of etanercept significantly decreases the secretion of the chemokine CXCL10 from MDM stimulated with *A. fumigatus* (p = 0.0004, Fig. 4). Furthermore, our data suggest that patients with IA being treated etanercept have lower CXCL10 serum concentrations compared to IA patients without treatment (Fig. 4). All patients analysed suffered from IA relatively late after alloSCT (range 108–219 days) and IA occurred during or immediately after administration of etanercept.

It is well established that high levels of TNF-alpha activate CXCL10 secretion via the STAT1 – NFkB1 (p50) – RelA (p65) pathway^38^. CXCL10 in blood and tissue exhibits strong chemotactic properties for monocytes, Natural Killer cells and CD4 + T cells leading to the conclusion that decreased CXCL10 levels prevent the presence of a variety of immune cell populations important for successful defense of IA^39,40^. Furthermore, Zhao and colleagues recently demonstrated a direct role of CXCL10 in IFN-γ-primed human monocytes to induce a robust production of proinflammatory cytokines, such as IL-12 and IL-23^41^. This leads to the conclusion that reduced concentrations of CXCL10 (in addition to already markedly reduced TNF-alpha concentrations due to etanercept treatment) might multimodally hamper a successful defense against *A. fumigatus* in patients treated with etanercept.

In this context, our own group demonstrated in 2008 that healthy individuals have lower CXCL10 concentrations in sera in comparison to patients after alloSCT. Furthermore, alloSCT patients with no fungal infection showed lower CXCL10 concentrations in comparison to patients with proven or probable IA, being highest in those patients who survived IA^42^. Moreover, SNPs in *CXCL10* were described to be associated with a reduced expression of *CXCL10* and an increased risk of IA^42^, which again shows the comprehensive functional relevance of this chemokine during *Aspergillus* infections.

Taken together, our *in vitro* studies show the ability of etanercept to inhibit immune responses through modulation of NFkB activity, TNF-alpha activity and CXCL10 release, suggesting that etanercept treatment is an important risk factor for the development of IA.

In conclusion, our study provides new and valuable information on potential factors predicting the risk for IA in alloSCT recipients. They also provide mechanistic and functional details on how etanercept treatment lowers CXCL10 concentrations *in vitro* and *in vivo*, thereby multiplying the effect of already diminished TNF-alpha concentrations. This study should direct clinicians to the fact that low monocyte counts and the administration of etanercept necessitate special caveats, such as continuous monitoring of patients with highly sensitive diagnostic assays and the use of anti-*Aspergillus* prophylaxis. Further large multi-centre studies will be necessary to identify new biomarkers and to evaluate existing markers on a broader basis considering multi-factorial and multimodal analysis strategies.

## Materials and Methods

### Characterization of clinical variables

A collection of 96 static and seven dynamic, i.e. temporal, variables were defined previously (Supplementary Table 1) to acquire patient characteristics in the frame of the study “Aspergillosis – Individual Risk Estimation (ASPIRE, unpublished data). The static variables included age, gender, underlying disease, stem cell type, treatment variables such as the use of antibiotics, antifungal drugs, and drugs for immune suppression. Temporal variables comprised blood cell counts including total leukocytes, monocytes, granulocytes and lymphocytes.

### Selection of patients

Between 2007 and 2013, patients undergoing alloSCT at the University Hospital of Würzburg were followed and categorized according to the first revision of the EORTC/MSG definitions^6^. Among them, a total of 36 patients with probable IA yielded complete clinical data records containing all 103 hitherto defined variables mentioned above. Thus, these patients qualified for inclusion into the current study.

Cases (with IA) and respective controls without signs of IA (n = 36, respectively) were balanced according to gender, age and underlying diseases (Table 1). All study patients suffered from defined haematological malignancies and subsequently underwent an alloSCT at the University Hospital Wuerzburg^43^.

The main underlying disease was acute myeloid leukemia (AML, n = 20) followed by multiple myeloma (n = 15) (patient demographics are shown in Table 1). The total patient cohort consisted of 25 females and 47 males with an average age of 55 years (range: 20–72 years). In female patients, AML was the main underlying disease in both cases (n = 5) and controls (n = 4), followed by other diseases (cases, n = 4; controls, n = 3), multiple myeloma (cases, n = 2; controls, n = 2), myelodysplastic syndrome (cases, n = 1; controls, n = 2), and acute lymphoblastic leukemia (cases, n = 1; controls, n = 1). In male patients, multiple myeloma (controls, n = 9) and AML (cases, n = 8) were the main underlying diseases, followed by myelodysplastic syndrome (cases, n = 3; controls, n = 2), chronic lymphocytic leukemia (cases, n = 3; controls, n = 2), acute lymphoblastic leukemia (cases, n = 4; controls, n = 1), and other diseases (cases, n = 3; controls, n = 7).

Controls did not show any signs of fungal infection. To study temporal changes after alloSCT, we collected all variables (Supplementary Table S3) twice weekly (when available) starting directly after SCT (day + 0) until the patient was discharged from the hospital or died. Patients developed IA on average 105 days after alloSCT.

To confirm the most relevant results of this study, a second, independent validation cohort was defined. For this second patient cohort, only information about significant variables identified by the first cohort was collected. Patients, within complete data for103 variables and consequently excluded from the first cohort were included in the validation cohort. Again, patients who suffered from hematological malignancies and received alloSCT between 2007 and 2015 were considered. This cohort consisted of 23 cases (with probable IA) and 25 controls, with no evidence of fungal infection. The cohort comprised 25 females and 23 males with an average age of 56 years (range: 26–72 years). The main underlying disease was AML (n = 28) followed by multiple myeloma (n = 5). The average time from alloSCT to IA diagnosis was 101 days.

### Blood cell counts

Quantitative data on blood cell counts from cases were obtained weekly over a four week window prior to the diagnosis of IA, using the date of diagnosis of IA as a reference time point. Suitable controls were matched considering only samples with a similar temporal distance to the date of alloSCT as a reference.

### *In vitro* generation of human monocyte-derived macrophages (MDM)

Peripheral blood mononuclear cells (PBMC) were isolated from three healthy anonymous volunteers (Institute of Transfusion Medicine and Haemotherapy, University Hospital Würzburg) by density gradient centrifugation using Biocoll separating solution (Biochrom). Monocytes were purified from PBMCs by magnetic cell sorting using CD14 microbeads (Miltenyi Biotech) and incubated at 37 °C and 5% CO_2_ atmosphere in RPMI-1640 supplemented with 10% heat-inactivated FCS, 120 µg/ml Refobacin (Merck) and 25 ng/ml GM-CSF (Sanofi-Aventis). MDM were used at day six for further analyses.

### Aspergillus fumigatus culture

*Aspergillus fumigatus* ATCC 46645 (American Type Culture Collection) was used as a stimulus for all experiments. Cultivation of *A. fumigatus* and generation of germ tubes was according protocol described by Hefter and collegues^44^. After overnight incubation, germ tubes were centrifuged for 5 minutes at 5000xg and resuspended in fresh medium. MDM were incubated for 6 hours with live *A. fumigatus* germ tubes at a multiplicity of infection (MOI) of 0.5 at 37 °C and 5% CO_2_ atmosphere.

### Etanercept

The effects of etanercept (Enbrel®, Pfizer, Germany) on macrophage gene expression profiles and cytokine response were evaluated at a concentration of 2 µg/ml, which is comparable with concentrations found in plasma of arthritic patients^45,46^. MDM were challenged with etanercept for 6 hours at 37 °C in a 5% CO_2_ atmosphere.

### RNA extraction, RNA sequencing and RNAseq data processing

The cell pellet was resuspended in RNAprotect (Qiagen) and RNA was extracted using the RNeasy mini kit (Qiagen) according to the manufacturer’s protocol. RNA concentration and purity were determined in a NanoDrop ND-1000 spectral photometer (Thermo Fisher Scientific). The integrity of all samples was analysed in a 2100 Bioanalyzer (Agilent Technologies, Germany) using the RNA 6000 Nano LabChip Kits according to the manufacturer’s instructions (Agilent Technologies). All samples had RIN values above 8.5 and were sent for library preparation, rRNA removal, and 75 bp single-end sequencing on an Illumina Next Seq 500 to IMGM Laboratories GmbH (Munich, Germany). Raw sequencing reads were deposited at Gene Expression Omnibus (GSE113254). Reads were quality checked using FastQC and trimmed using Trimmomatic (v 0.36, window size 15 average quality 25, MINLEN:30)^47^. Quality trimmed reads were mapped onto the human reference genome (GRCh38) using HiSat2 (v 2.1.0) by supplying GFF file^48^. A number of reads were counted using the Rsubread package^49^ together with the gene structural annotation (GRCh38-GFF file). Raw counts were normalized using RPKM values. Differentially expressed genes were detected using DESeq2^50^ with an adjusted p-value of ≤ 0.05 combined with an absolute log2-fold-change cutoff of at least 1.5. Enriched Gene Ontology categories were detected using the tool FungiFun2^51^. The following genes were further analysed in a gene regulatory network: BCL3 (ENSG00000069399), BIRC3 (ENSG00000023445), CXCL10 (ENSG00000169245), ICAM1 (ENSG00000090339), NFKB1 (ENSG00000109320), and RELB (ENSG00000104856). Network interactions were derived from the database of Pathway Studio V9, which contains automatically extracted information from PubMed publications^52^.

### Cytokine quantification

The abundance of cytokines secreted by MDM was measured using a Bio-Plex Pro Human Cytokine 27-plex Assay (Bio-Rad). CXCL10 concentration in serum samples (n = 80) from IA patients (n = 8) with or without etanercept treatment was measured with the ELISA MAX^TM^ - human CXCL10 (IP-10) assay (BioLegend) according to the manufacturer’s instructions. Preprocessing of the data was performed according to the instructions from the manufacturer. Differentially secreted cytokines were detected using Wilcoxon-Mann-Whitney tests applying a p-value ≤ 0.05.

### Statistical methods

Univariate logistic regression models were employed to find associations between all variables and the diagnosis of IA (case-control status) in the training data set of 36 patients suffering from probable IA and 36 control patients. For temporally dynamic variables, models were generated separately for each week and the first 4 weeks before diagnosis. Since blood cell counts can vary considerably over time, we performed time-dependent case-control matching. For example, the monocyte concentration of a case suffering from IA at day 60 after alloSCT was compared with a matched monocyte concentration of a control patient also (or closest to) 60 days after alloSCT. Each model and thus the contribution of each variable were evaluated by a likelihood ratio test (LRT) against an intercept-only model. We explored the univariate models and pursued variables if their univariate screening p-value was ≤ 0.05. Variables with too little variability were not considered further. Afterwards, multiple logistic regression models were built to test the joint combined effect of the selected variables from the univariate models. Additional variables were integrated and tested with LRT for improvement of the prediction power of the model.

The predictive power of our final model was assessed using an independent, similarly ascertained, validation sample of 23 IA cases and 25 control patients. We report statistical estimates of the area under ROC curve (AUC) for all models to allow for evaluation of the model. All statistical analyses were done in the statistical environment R (version 3.3).

### Ethics approval

All methods were performed in accordance with the relevant guidelines and regulations. Informed consent has been obtained for study participation and for publication of information, as requested by the Ethics Committee of the University Hospital of Wuerzburg (permit #173/11).

## Supplementary information

Supplementary material
