# Supplementary material for: Treatment with etanercept and low monocyte concentration contribute to the risk of invasive aspergillosis in patients post allogeneic stem cell transplantation
Source: Sci Rep. 2019 Nov 21;9:17231. doi: 10.1038/s41598-019-53504-8 (PMC6872713; doi:10.1038/s41598-019-53504-8)
Supplement: Supplementary file 1 — Supplementary material [file 41598_2019_53504_MOESM1_ESM.pdf]

Treatment with etanercept and low monocyte concentration contribute to the risk of invasive aspergillosis in patients post allogeneic stem cell transplantation

Tamara Zoran*, Michael Weber*, Jan Springer, P. Lewis White, Joachim Bauer, Annika Schober, Claudia Löffler, Bastian Seelbinder, Kerstin Hünniger, Oliver Kurzai, André Scherag, Sascha Schäuble, C. Oliver Morton, Hermann Einsele, Jörg Linde[#], Jürgen Löffler[#,§]

Supplementary material

*,# Both authors contributed equally

§Correspondence:
Prof. Dr. Jürgen Löffler
University Hospital Würzburg,
Medical Hospital II, WÜ4i, Building C11, 97080 Würzburg, Germany
Telephone +49 931 20136412, Fax: +49 931 20136409
Loeffler_j@ukw.de

Supplemetary Table S1: Comparison of cytokine response between:
a) unstimulated monocyte-derived macrophages (MDM) and MDM stimulated with *A. fumigatu*s,
b) unstimulated MDM and MDM stimulated with etanercept,
c) MDM stimulated with *A. fumigatus* and MDM stimulated with *A. fumigatus* and etanercept.
MDM from 3 donors were stimulated with *A. fumigatus* at 37°C for 6 hours.

	Cytokine	Concentration (pg/ml)		
		**unstimulated MDM**	**MDM stimulated with AF**	**p-value**
a)	FGF	0.00	63.38	0.0116
	IL9	28.86	474.92	0.0136
	IFNG	17.27	144.11	0.0166
	Eotaxin	0.0	84.71	0.0198
	IL17	0.0	171.34	0.0206
	IL6	10.32	580.14	0.0233
	TNFalpha	14.75	1365.21	0.0286
	MIP1beta	267.19	8614.88	0.0331
	IL8	523.67	13425.00	0.0416
	CXCL10			0.0452
		**unstimulated MDM**	**MDM stimulated with ET**	
b)	MCP1	284.47	374.50	0.00788
	IL1RA	9850.4	12335.72	0.0424
		**MDM stimulated with AF**	**MDM stimulated with AF and ET**	
c)	IP10	1624.28	248.23	0.000369
	TNFalpha	1365.21	440.78	0.0104
	GCSF	19.16	15.30	0.035

Supplemetary Table S2: Comparison of gene expression between monocyte-derived macrophages (MDM) stimulated with *A. fumigatus* and MDM stimulated with *A. fumigatus* and etanercept. MDM from 3 donors were stimulated with *A. fumigatus* and with or without etanercept at 37°C for 6 hours.

Gene	Name	ID	Log2Fc	p-value
MT1M	metallothionein 1M [Source:HGNC Symbol;Acc:HGNC:14296]	ENSG00000205364	-Inf	0.00016113
STK17A (DRAK1)	serine/threonine kinase 17a [Source:HGNC Symbol;Acc:HGNC:11395]	ENSG00000164543	-0.4691	0.04276152
ICAM1	intercellular adhesion molecule 1 [Source:HGNC Symbol;Acc:HGNC:5344]	ENSG00000090339	-0.5299	0.04834824
BID	BH3 interacting domain death agonist [Source:HGNC Symbol;Acc:HGNC:1050]	ENSG00000015475	-0.5719	0.03493310
FUT4	fucosyltransferase 4 [Source:HGNC Symbol;Acc:HGNC:4015]	ENSG00000196371	-0.6437	0.00011280
RELB	RELB proto-oncogene, NF-kB subunit [Source:HGNC Symbol;Acc:HGNC:9956]	ENSG00000104856	-0.7057	0.00648974
RASGRP1	RAS guanyl releasing protein 1 [Source:HGNC Symbol;Acc:HGNC:9878]	ENSG00000172575	-0.8440	0.04276152
RNF144B	ring finger protein 144B [Source:HGNC Symbol;Acc:HGNC:21578]	ENSG00000137393	-0.8847	0.00000005
POU2F2	POU class 2 homeobox 2 [Source:HGNC Symbol;Acc:HGNC:9213]	ENSG00000028277	-0.9547	0.00156170
BIRC3	baculoviral IAP repeat containing 3 [Source:HGNC Symbol;Acc:HGNC:591]	ENSG00000023445	-1.2690	0.00039248
SLC2A6	solute carrier family 2 member 6 [Source:HGNC Symbol;Acc:HGNC:11011]	ENSG00000160326	-1.3496	1.58787E-10
BCL3	B-cell CLL/lymphoma 3 [Source:HGNC Symbol;Acc:HGNC:998]	ENSG00000069399	-1.3657	0.048348243
MT2A	metallothionein 2A [Source:HGNC Symbol;Acc:HGNC:7406]	ENSG00000125148	-2.0620	5.00529E-17
MT1E	metallothionein 1E [Source:HGNC Symbol;Acc:HGNC:7397]	ENSG00000169715	-2.3266	3.3364E-20
MT1F	metallothionein 1F [Source:HGNC Symbol;Acc:HGNC:7398]	ENSG00000198417	-2.3441	8.8808E-16
MT1X	metallothionein 1X [Source:HGNC Symbol;Acc:HGNC:7405]	ENSG00000187193	-3.2504	2.98045E-17
MT1G	metallothionein 1G [Source:HGNC Symbol;Acc:HGNC:7399]	ENSG00000125144	-4.0949	2.08693E-30
MT1H	metallothionein 1H [Source:HGNC Symbol;Acc:HGNC:7400]	ENSG00000205358	-4.3569	2.36785E-09

Supplemetary Table S3: Collection of dynamic and binary clinical variables obtained from alloSCT patients at University hospital Würzburg.

Variables			
loperamide	galactomannan	sex	alkylsulfonates: Busulphan, Teosulfan
nitroimidazoles	neutropenia >10d	summer	ATG
fluorchinolone	Epstein Barr virus	fall	thiotepa
acylaminopenicillines	*Staphylococcus epidermidis*	winter	total body irradiation
betalactamase-inhibitors	human herpes virus 6	spring	allogenic, bone marrow transplantation, umbilical cord blood transfusion
cephalosporines	cytomegalovirus	T cell immunosuppression in last 90d	matched, unmatched
carbapenemes	other_pathogens_than_aspergillus	sirolimus	acute myeloid leukemia
amphotericin B	gram-negative rods	mycophenolatmofetile	myelodysplastic syndrome
fluconazole	*Candida albicans*	antithymocyte globulin (Genzyme)	acute lymphoblastic leukemia
voriconazole	*Escherichia coli*	prednisolone, dexamethasone, hydrocortisone, fludrocortisone	chronic lymphocytic leukemia
posaconazole	*Klebsiella*	muromonab	multiple myeloma
caspofungin	*Proteus*	etanercept	plasmocytoma
anidulafungin	viridans streptococci	budesonide	
Aspergillus PCR	herpes simplex virus 1	T-cell immunosuppression	
dexamethasone	*Clostridium difficile*	nucleoside analogues: valganciclovir, ganciclovir, foscavir, cidofovir, maribravir (anti-cytomegalovirus drugs)	
hydrocortisone	Varicella-zoster virus	anti herpes simplex virus: aciclovir, brivudin, famciclovir, valaciclovir	
prednisolone	vancomycin resistent enterococci	degree of GvHD	
corticosteroide >21d >0,3mg/kg KG/d	polyomavirus	arterial hypertension	
corticosteroids_yes_no	*Candida Krusei*	thomboembolic events: deep vein thombosis, vena jugularis interna thrombosis, carotid endarterectomy, pulmonary embolism	
betamethasoncreme	toxoplasmosis	renal diseases: acute renal failure, chronic renal insufficiency, hydronephrosis, nephrolithiasis	
dexamethasone	*Candida glabrata*	heart diseases: cardiac arrhythmia, patent foramen ovale, mycardial infarction, left ventricular insufficiency, coronary heart disease	
hydrocortisone	gram-positive coccal bacteria	drug incompatibility	
prednisolone	Coagulase-negative Staphylococci	neurological diseases: migraine, depression, gait ataxia, polyneuropathy, cluster headache, multiple sclerosis, hemiplegia, artery cerebri media infarction	
budesonide	*Enterococcus faecalis*	orthopaedic diseases: osteoporosis, knee operation, fractures, lumbal spinal stenosis, avascular necrosis of the femoral head	
creatinine normal	immunglobulins	purine analogues:Fludarabin, Mercaptopurin, Nelarabin, Tioguanin, Clofarabin	
human albumin	mouth wash	nitrogen mustard analogues: Bendamustin, Cyclophosphamid, Chlorambucil, 42, Ifosfamid, Trofosfamid	
etanercept	age		
